# Associating pancreaticostomy and biliary-irrigation for staged pancreaticoduodenectomy approach to pancreatic intraductal papillary mucinous neoplasm with recurrent cholangitis and severe jaundice: A case report

**DOI:** 10.1097/MD.0000000000005500

**Published:** 2016-12-02

**Authors:** Chao Dai, Siyuan Lou, Fan Zhou

**Affiliations:** a Department of Hepatobiliary and Pancreatic Surgery, the Second Affiliated Hospital of Nanchang University; b Jiangxi Provincial Center for Hepatobiliary Disease, Nanchang, China.

**Keywords:** cholangitis, intraductal papillary mucinous neoplasm (IPMN), jaundice

## Abstract

**Patient concerns:**

A 63-year-old man was hospitalized with history of abdominal pain since more than 1 year, and that of fever with chills since 2 weeks.

**Diagnoses:**

Based on the laboratory investigations and radiologic findings, a preliminary diagnosis of pancreatic intraductal papillary mucinous neoplasm (IPMN) with recurrent cholangitis and severe jaundice was made.

**Interventions:**

An initial attempt at endoscopic and image-guided drainage proved unsuccessful. Due to cholangitis, liver dysfunction, and hypoalbuminemia, the patient was deemed to be medically unfit for radical surgery. Therefore we considered a novel strategy of associating pancreaticostomy and biliary-irrigation for staged pancreaticoduodenectomy (APBSP). In the first stage, biliary tract double irrigation (endoscopic nasobiliary drainage and T-tube) in combination with pancreaticostomy was performed, which alleviated the symptoms and helped improve the general condition of the patient. In the second stage, radical pancreaticoduodenectomy was performed.

**Outcomes:**

Over a follow-up period of 23 months, no recurrence occurred.

**Lessons:**

In this report, we present a previously unreported treatment strategy for pancreatic IPMN with recurrent cholangitis and jaundice. The innovative treatment approach may help advance the understanding and management of this condition.

## 1 Introduction

Intraductal papillary mucinous neoplasm (IPMN) of the pancreas is an exocrine tumor composed of intraductal papillary growth of mucin containing neoplastic cells in the main pancreatic duct or its branches.^[1]^ IPMN encompasses a wide spectrum that ranges from adenomas to invasive cancer with various degrees of aggressiveness.^[2]^ Infrequently, IPMN can invade into the adjoining organs including duodenum (59–64%), common bile duct (CBD) (51–57%), and stomach (17%).^[3]^

## 2 Case report

A 63-year-old man was hospitalized with a 2-week-long history of fever with chills. He had a history of abdominal pain since more than 1 year, and had lost about 15 kg in weight over the preceding 1 year. There was no other significant past medical or surgical history. On general physical examination, the patient showed signs of jaundice with upper abdomen tenderness but no guarding. Liver panel showed highly elevated total bilirubin (TBIL, 232.18 μmol/L, normal reference [NR]: 1.71–17.10 μmol/L) and direct bilirubin (DBIL, 192.17 μmol/L, NR: 1.71–7.00 μmol/L). Total white blood cell (WBC) count was 10.60 × 10^9^/L (NR: 4.00–10.00 × 10^9^/L) and the percentage of neutrophils was 72% (NR: 50–70%). Serum albumin was decreased (27.75 g/L [NR: 38.00–55.00 g/L]), while carbohydrate antigen 19-9 level was elevated (753.50 U/mL [NR: 0.00–40.00 U/mL]).

**Figure 1:**
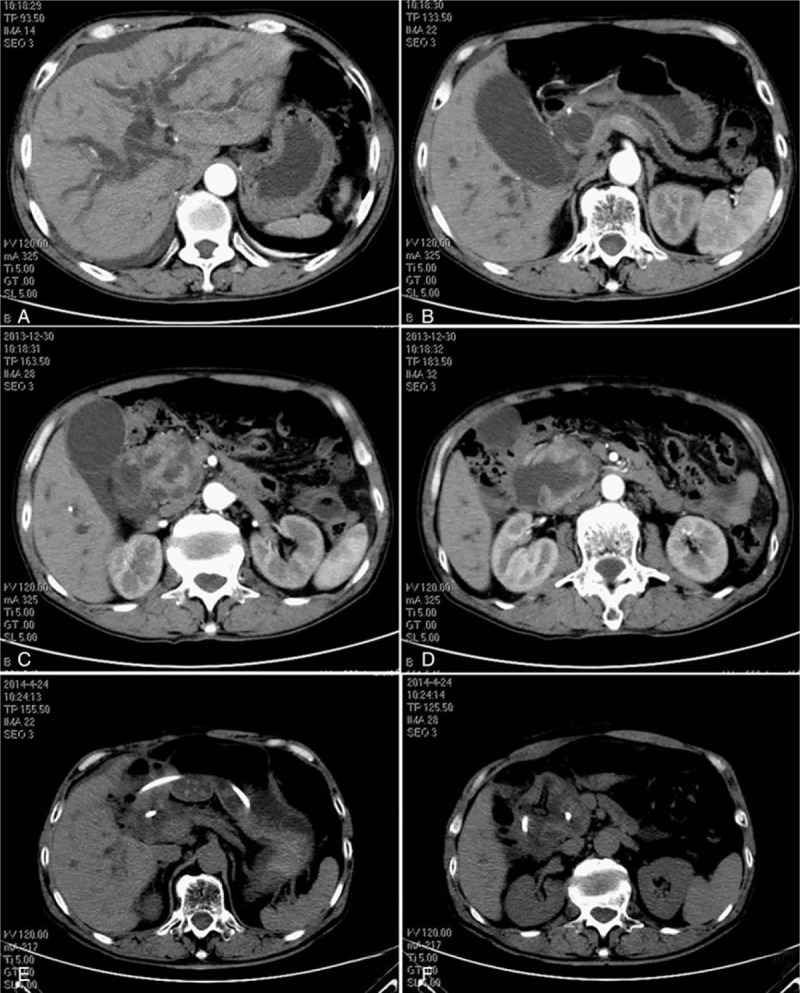
(A and B) Contrast-enhanced CT scan showed marked dilatation of intrahepatic and extrahepatic bile ducts in the absence of any luminal filling defect, main pancreatic duct of size >10 mm and signs of distal pancreatic atrophy. (C and D) A heterogeneous, cystic mass measuring 4.2 × 5.4 cm involving the duodenum and pancreatic head with thickened enhanced cyst walls, with an abnormal communication between the mass and the descending part of the duodenum. (E and F) Obstruction of biliary and pancreatic duct was significantly decreased after associating pancreaticostomy and biliary-irrigation.

Contrast-enhanced computed tomography (CT) revealed a 4.2 × 5.4 cm, heterogeneous mass involving the duodenum and pancreatic head with enhancing wall (Fig. 1A–D). On endoscopic retrograde cholangiopancreatography (ERCP) a viscid mucus flow through the large duodenal papilla was observed.

Empirical treatment with piperacillin/tazobactam was administered for suspected acute cholangitis. However, 3-day course of piperacillin/tazobactam did not produce the desired clinical effect. Antibiotic agent was changed to imipenem for 1 week. In order to reduce jaundice and control cholangitis, endoscopic nasobiliary drainage (ENBD) was performed. However, he did not respond to treatment. TBIL and DBIL continued to increase (461.99 and 385.17 μmol/L, respectively), accompanied by highly elevated WBC counts (23.17 × 10^9^/L) and percent neutrophils (85.7%). *Escherichia coli* was isolated from blood and bile cultures.

Based on the laboratory investigations and radiologic findings, a preliminary diagnosis of pancreatic IPMN was made. Due to cholangitis, liver dysfunction, and hypoalbuminemia, the patient was deemed unfit to undergo radical surgery. Here, we present a novel concept of associating pancreaticostomy and biliary-irrigation for staged pancreaticoduodenectomy (APBSP). The first-stage operation was biliary exploration with cholecystectomy, along with pancreaticostomy to ensure unobstructed flow of mucus at its source. The patient was instructed to regularly irrigate biliary tract via ENBD and T-tube. This was continued for 3 months at the local community hospital, and successful biliary decompression was achieved (Fig. 1E and F). TBIL and DBIL showed a significant decrease (30.22 and 25.35 μmol/L, respectively). WBC and percent neutrophils were restored to normal. Subsequently, the patient underwent pancreaticoduodenectomy and a final diagnosis of pancreatic IPMN invading the duodenum was established. The patient has since been living in good health with no symptoms of the disease for 23 months.

The study protocol was established, according to the ethical guidelines of the Helsinki Declaration and was approved by the Human Ethics Committee of The Second Affiliated Hospital of Nanchang University, China. Written informed consent was obtained from individual participant.

## 3 Discussion

Biliary obstruction frequently develops during the course of pancreatic IPMN. Biliary drainage is often achieved by ERCP, percutaneous transhepatic cholangiography (PTC), ultrasound (US)-guided drainage, or surgical drainage. The present case highlights the difficulty in providing sustained biliary drainage by endoscopic methods.

Although the intrahepatic bile ducts were dilated, we did not perform PTCD or resort to biliary stenting in the present case. There were several reasons for adopting this strategy. First, jaundice was a consequence of direct compression by tumor and/or biliary obstruction by viscid mucin accumulation.^[4]^ In the former case, patients tend to respond well to ENBD and percutaneous transhepatic cholangial drainage (PTCD).^[5]^ However, in the latter situation, PTCD is likely to be ineffective due to rapid occlusion by thick mucin. In our previous experience with PTCD in similar cases, we often encountered obstruction of tube, which necessitated repeat procedures. Secondly, cholangitis from pancreatic IPMN is typically hard to treat by percutaneous drainage. This was also illustrated by the case in Kurihara's report^[6]^ who died of cholangitis despite percutaneous transhepatic catheter drainage.

In a report by Patel et al,^[7]^ the mucinous material was cleared by balloon extraction and biliary stents were repeatedly placed. Despite these efforts, jaundice continued to worsen and was accompanied by recurrent cholangitis. Hence, we believed that multiple attempts at endoscopic or radiological management of these patients should be avoided. The poor general condition of the patient was a key challenge, and the treating team felt that a surgical approach might offer the chance for successful biliary drainage for sustained periods of time.

Therefore we considered a novel APBSP approach in this case. As part of the first stage, biliary tract double irrigation (ENBD and T-tube) in combination with pancreaticostomy was performed. Pancreaticostomy ensured unobstructed flow of mucus at its source. The role of double irrigation by ENBD and T-tube was to maintain decompression and flow in the biliary tract. This combination proved effective in alleviating symptoms and improving the general condition of the patient. In the second stage, radical pancreaticoduodenectomy was undertaken. The interval between the two stages depends on the state of abdominal infection and subsidence of biliary inflammation.

In this report, we presented a novel treatment strategy for pancreatic IPMN with recurrent cholangitis and severe jaundice. APBSP strategy should be considered in similar cases.
